# Think Outside the Box: A Rare Presentation of Schistosomiasis in the United States

**DOI:** 10.7759/cureus.27970

**Published:** 2022-08-13

**Authors:** Humayun Anjum, Farah Yasmin, Syed Hasan Ali, Shaleen Sunesara, Aiza M Khawaja, Annie Haji Datoo, Mohammed Ali, Salim Surani

**Affiliations:** 1 Pulmonary and Critical Care Medicine, Baylor Scott & White Health, Dallas, USA; 2 Internal Medicine, Dow University of Health Sciences, Karachi, PAK; 3 Population and Public Health, University of Southern California, Los Angeles, USA; 4 High School, Tompkins High School, Houston, USA; 5 Internal Medicine, St. Joseph's Medical Center, Stockton, USA; 6 Pulmonary and Critical Care Medicine, Corpus Christi Medical Center, Corpus Christi, USA; 7 Anesthesiology, Mayo Clinic, Rochester, USA; 8 Medicine, Texas A&M University, College Station, USA; 9 Medicine, University of North Texas, Dallas, USA; 10 Internal Medicine, Pulmonary Associates, Corpus Christi, USA; 11 Clinical Medicine, University of Houston, Houston, USA

**Keywords:** eosinophilia, diarrhea, tropical illness, abdominal pain, flatworms, pancreatitis, schistosomiasis

## Abstract

Millions of people across the world are infected with schistosomiasis. But, the vast majority of them are asymptomatic. Milder symptoms can include headache, lethargy, and fever. In serious cases, ascites, hepatosplenomegaly, and death can occur. Schistosomiasis is a highly prevalent parasitic infection worldwide, mainly in tropical areas of Africa, Asia, and Latin America. We present a case of a 69-year-old female with notable travel history to the Philippines, who reported to the emergency department with a symptomatic presentation of chronic schistosomiasis with the involvement of biliary and pancreatic ducts.

## Introduction

Schistosomiasis is a disease caused by the trematode *Schistosoma* of the flatworms’ phylum. Platyhelminthes remains an uncommon presentation in developed countries, including the United States. The disease's incidence rate depends primarily on two factors: the population of the species' intermediate hosts (various types of freshwater snails) and the degree of human waste contamination in freshwater bodies [1,2]. Consequently, it is predominantly encountered in tropical and subtropical areas, mainly Sub-Saharan Africa and some South American countries. However, cases sporadically appear in various other regions, likely due to increased travel and migration from areas of high prevalence, as mentioned earlier. Statistics show that around 240 million people required preventive treatment for the disease in 2019 [2].

Schistosomiasis is acquired through the skin when humans come in direct contact with freshwater sources containing the infective form (cercariae) of these blood flukes. It progresses in three phases: acute, chronic, and advanced [3]. The progression depends on the type of species involved and the intensity of the infection. However, the most common scenario or presentation is that patients remain asymptomatic, and only chronic conditions result in clinical and systemic manifestations. The chronic forms are categorized into two main types based on the pathogenesis and eventual clinical presentation of the disease: intestinal and urogenital schistosomiasis. Intestinal schistosomiasis is caused mainly by *Schistosoma mansoni* and *Schistosoma japonicum*, while *Schistosoma mekongi* and *Schistosoma guineensis* also play a role in the incidence of the disease. The urogenital form is the result of *Schistosoma haematobium* [2,4].

Here, we present a case of schistosomiasis in the United States with a unique presentation of biliary and pancreatic ductal involvement.

## Case presentation

A 69-year-old female with a history of hypertension and osteoarthritis presented to the emergency department (ED) of a local hospital in Texas. She complained of abdominal pain, diarrhea, and yellow skin discoloration. The abdominal pain had been intermittent for the last few months, but diarrhea started only a few days prior to the presentation. The patient also had a notable recent travel history to the Philippines about eight months prior to the presentation to the ED. On examination, she was noted to have tenderness in the right upper abdominal quadrant with hepatomegaly. She was afebrile and had no appreciable skin rashes or lesions. Laboratory findings are mentioned in Table 1.

**Table 1:** Laboratory findings BUN: blood urea nitrogen; SGOT: serum glutamic-oxaloacetic transaminase; AST: aspartate aminotransferase; SGPT: serum glutamic pyruvic transaminase; ALT: alanine aminotransferase.

Laboratory parameter	Laboratory results	Reference range
WBC	13.6 K/uL	4.5-11.0 K/uL
RBC	4.13 M/uL	4.50-6.0 M/uL
Hemoglobin	11.5 g/dL	12.0-16.0 g/dL
Hematocrit	34.3%	36.0-47.0%
Platelet count	282 K/uL	140-440 K/uL
Segmented neutrophils	37%	N/A
Basophils	1%	N/A
Eosinophils	6%	N/A
Glucose	132 mg/dL	70-99 mg/dL
BUN	28 mg/dL	7-18 mg/dL
Creatinine	1.18 mg/dL	0.55-1.02 mg/dL
Sodium	138 meq/L	136-145 meq/L
Potassium	3.6 meq/L	3.5-5.1 meq/L
Chloride	101 meq/L	98-107 meq/L
Carbon dioxide	28 meq/L	21-32 meq/L
Calcium	7.9 mg/dL	8.5-10.1 mg/dL
Bilirubin, total	7.5 mg/dL, peak 17.9 mg/dL	0.2-1.0 mg/dL
Alkaline phosphatase	116 U/L	45-117 U/L
SGOT (AST)	198 U/L	15-37 U/L
SGPT (ALT)	182 U/L	13-56 U/L
Protein, total	6.9 g/dL	6.4-8.2 g/dL
Albumin	2.8 g/dL	3.4-5.0 g/dL
Lipase	1005 U/L	73-393 U/L
Immunoglobulin E, quantitative	622 IU/ml	1.5-144 IU/ml
Ova and parasites, stool	Negative including Kato-Katz smear	N/A

**Figure 1:**
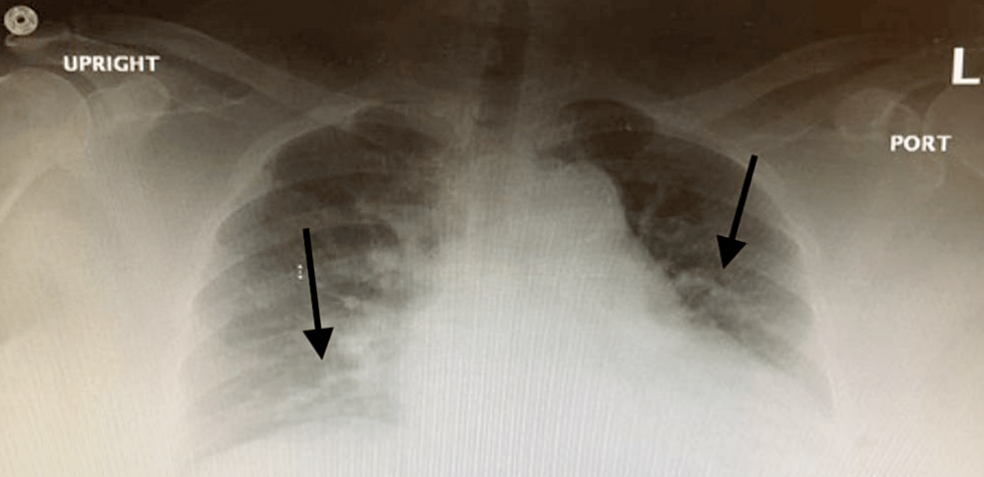
Chest X-ray anteroposterior view showing increased vascular markings

Laboratory workup was remarkable for leukocytosis with mild eosinophilia, elevated immunoglobulin E levels, elevated total bilirubin, aspartate aminotransferase (AST), alanine aminotransferase (ALT), and lipase levels. Chest X-ray showed increased vascular markings (Figure 1).

**Figure 2:**
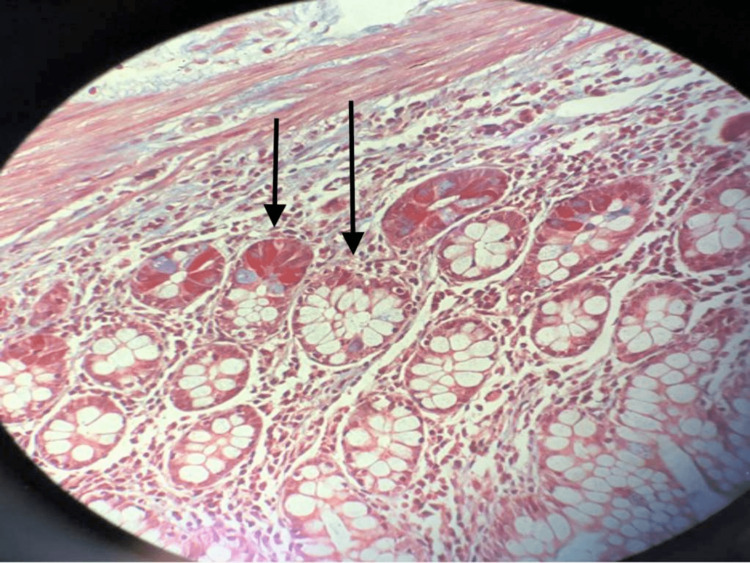
Schistosomiasis with calcified eggs, microvesicular steatosis, and portal fibrosis

Radiological findings from abdominal ultrasound and MRI revealed cholelithiasis, thickened gallbladder wall, trace pericholecystic fluid, and no biliary duct dilatation. Endoscopic retrograde cholangiopancreatography (ERCP) showed poor biliary duct visualization due to strictures. Brush biopsies were obtained, and a common bile duct stent was placed. A liver biopsy was subsequently performed. Pathology from the liver biopsy confirmed schistosomiasis with calcified eggs, microvesicular steatosis, and portal fibrosis (Figure 2). With the diagnosis in hand, treatment with praziquantel 60 mg/kg was started immediately in the hospital with a repeat dose in two weeks as an outpatient.

The patient was seen as an outpatient with repeat labs and was noted to have almost complete resolution of her symptoms except for some intermittent but significantly improved abdominal pain. Tender hepatomegaly resolved. Her total bilirubin was mildly elevated at 2.5 mg/dL. A repeat dose of 40 mg/kg was arranged. At the last follow-up visit, the patient was completely asymptomatic and was noted to have normalization of her bilirubin levels.

## Discussion

This case shows a clinical picture closely related to intestinal-hepatic schistosomiasis. The symptomatic manifestations usually include and progress from abdominal pain, hepatomegaly, and/or splenomegaly to hepatic and periportal fibrosis, portal hypertension, and esophageal and/or gastric varices [5]. The exsanguination from these ruptured varices is the most common cause of death in advanced cases [2]. The pathologic findings of this form of chronic schistosomiasis primarily arise due to the host's T lymphocyte-mediated immune response to the antigen secreted by schistosome eggs rather than the flatworm itself. The eggs of *S. mansoni* and *S. japonicum* most likely reside in hepatic and mesenteric circulation, the continuous inflammation of which can promote the formation of granulomas and subsequent hepatic fibrosis without endangering liver function [5].

Periportal fibrosis, the characteristic finding of schistosomiasis, can be well visualized with the help of ultrasonography and MRI [3]. Ultrasound imaging in specific can also help establish the extent of hepatic and splenic involvement in the disease and is a widely recognized and accurate parameter for assessing the morbidity due to chronic infection in clinical settings [3,6]. The findings of increased thickness of the gallbladder wall and trace pericholecystic fluid in our patient indicate inflammatory etiology, which most definitely could be a direct cause of the immune response, as stated earlier. Furthermore, with the evident finding of periportal fibrosis, we also noted the involvement of biliary and pancreatic ducts, which is a rare occurrence in patients with schistosomiasis. A study involving a murine model of *S. mansoni* infection revealed alterations of bile ducts with simultaneous ductular proliferation in chronic stages of infection, likely due to a secondary reaction to the increased number of eggs and/or granulomas [7]. Though there is scarce literature on the pathophysiology behind the involvement of biliary and pancreatic ductal injury, it is an important histopathological finding that, if noted in other patients with hepatic inflammation and injury, should be tested for schistosomiasis. Ideally, the Kato-Katz smear in the stool should be repeated at least twice, which was done only once in this case.

The choice of treatment in the case of schistosomiasis, as is the case for numerous parasitic infections, is praziquantel. The label dosage of 60 mg/kg was administered at the hospital following diagnosis, with a repeat dose in two weeks. Evidence suggests that repeated dose treatment has a greater cure rate and egg reduction than a single standard dose [8].

## Conclusions

The incidence of schistosomiasis in the US is uncommon, and the finding of biliary and pancreatic duct strictures due to the disease is also very rare in general, with only a handful of cases ever being published with similar presentations. Therefore, this case report highlights the importance of having schistosomiasis as a differential, especially with a travel history to an endemic area.
